# Untargeted UHPLC-TOF/MS Lipidomic Analysis for the Investigation of Egg Yolks after Xylanase Supplementation of the Diet of Laying Hens

**DOI:** 10.3390/metabo13050649

**Published:** 2023-05-10

**Authors:** Artemis Lioupi, Georgios A. Papadopoulos, Domniki Gallou, Christina Virgiliou, Georgios I. Arsenos, Paschalis Fortomaris, Veerle Van Hoeck, Dany Morisset, Georgios Theodoridis

**Affiliations:** 1 Laboratory of Analytical Chemistry, School of Chemistry, Aristotle University of Thessaloniki, 54124 Thessaloniki, Greece; liouarte@chem.auth.gr (A.L.); domnikig95@gmail.com (D.G.);; 2 Biomic AUTh, Center for Interdisciplinary Research and Innovation (CIRI-AUTH), Balkan Center B1.4, 10th km Thessaloniki-Thermi Rd, 57001 Thessaloniki, Greece; 3 FoodOmicsGR Research Infrastructure, AUTh Node, Center for Interdisciplinary Research and Innovation (CIRI-AUTH), Balkan Center B1.4, 10th km Thessaloniki-Thermi Rd, 57001 Thessaloniki, Greece; 4 Laboratory of Animal Husbandry, School of Health Sciences, Faculty of Veterinary Medicine, Aristotle University of Thessaloniki, 54124 Thessaloniki, Greece; geopaps@vet.auth.gr (G.A.P.);; 5 School of Chemical Engineering, Aristotle University of Thessaloniki, 54124 Thessaloniki, Greece; 6 Kemin Europa N.V., Animal Nutrition and Health EMENA, 2200 Herentals, Belgium

**Keywords:** lipid profiling, xylanase supplementation, feed intervention, food, lipidomics, chemometrics, nutrition, LC/MS

## Abstract

Xylanase supplementation of diets is used to enhance nutrient digestibility in monogastrics which lack necessary enzymes for non-starch polysaccharide degradation. The effects of enzymatic treatment in the nutritional value of the feed are typically not comprehensively studied. Though the fundamental effects of xylanase on performance are well studied, limited data is available on the complex interactions between xylanase supplementation and hen physiology; therefore, the aim of this study was to develop a new, simple UPLC-TOF/MS lipidomics method for the analysis of hen egg yolks after supplementation with different amounts of xylanase. Sample preparation for the extraction of lipids was optimized and different sample preparation modes and solvent mixtures were tested. Optimal results for the extraction of total lipids were obtained by using the solvent mixture MTBE: MeOH (5:1, *v*/*v*). Multivariate statistical analysis of the signals of hundreds of lipids in positive and negative ionisation modes highlighted differences in several egg yolk lipid species-classes. Four lipid species-classes, phosphatidylcholines (PC and PC O), phosphatidylethanolamines (PE and PE O), phosphatidylinositols (PI), and fatty acids (FA), were among those contributing to the separation of the experimental groups (control-treated) in negative ionisation mode. In positive ionisation mode, principal beneficial lipid compounds such as phosphatidylcholines (PC and PC O), phosphatidylethanolamines (PE and PE O), triacylglycerols (TG), diacylglycerols (DG), and ceramides (Cer) were found to be increased in treated groups. Overall, supplementation of laying hens’ diets with xylanase significantly changed the lipid profile of egg yolks compared to the control diet. The association between the lipid profiles of egg yolks and hens’ diets, as well as the underlying mechanisms, require further investigation. These findings are of practical significance for the food industry.

## 1. Introduction

Eggs are an important source of bioactive components, including proteins and amino acids, vitamins, dietary lipids, and other substances. The lipid content of an egg yolk varies according to the hen’s feeding regime. Feed intervention can significantly alter the lipidomic profile of egg yolks, resulting in improved egg nutritional value and health benefits. Since poultry cannot efficiently digest dietary non-starch polysaccharides (NSPs), the addition of NSP-digesting enzymes to hens’ diets improves nutrient availability and bird performance [1].

Although the specific anti-nutritive activity of NSPs is unknown, it is thought that the mechanism of action involves encapsulation of dietary nutrients and/or increased intestinal digesta viscosity. More specifically, it has been shown that xylanase supplementation in laying hen diets consisting of corn, soybean, and dried distiller’s grain improved egg production and increased feed intake [2], and supplementation also in a wheat-rich diet improved egg production, while decreasing ideal digesta viscosity [3].

Xylanase is an enzyme that degrades long-chain arabinoxylans to small-chain xylo-oligomers, improving nutrient digestibility for animals and decreasing digesta viscosity correlated to high arabinoxylan intake [4]. Xylanase has been found to improve the digestibility of starch, fat, and protein, confirming the efficiency of this enzyme for degrading NSPs [4].

A comprehensive study of egg lipid content requires the use of accurate and sensitive analytical platforms that offer effective separation and detection of lipids and lipid species [5]. Liquid chromatography-tandem mass spectrometry (LC-MS/MS) offers increased throughput and confidence for lipidomics profiling studies. As a rule, high-resolution MS is the first option for lipidomic analysis, including analysers such as quadrupole-time-of-flight (Q-TOF), Orbitrap, and Fourier transform ion cyclotron resonance mass spectrometers [6,7,8]. Many LC configurations have been described for the chromatographic separation of neutral, semi-polar, and polar lipids from complex mixtures such as reversed phase (RP), normal phase (NP), and hydrophilic interaction chromatography (HILIC). Many studies have applied RPLC for the separation of polar and non-polar lipids in a single analysis, often using multistep gradient elution programs. Such methods use short (50–150 mm) columns with C18 or C8 sorbents.

Vorkas et al. [9] used an untargeted RP-UPLC-TOF-MS approach using simultaneous acquisition of exact mass at high and low collision energy (MS^E^) for the semi-quantitation of a wide range of lipid molecules (>250 lipids). For structural annotation of the detected lipids, the spectra from data-dependent acquisition modes were compared with spectra available from databases and the literature. Recently, the same type of column was applied in a lipidomics study to obtain a snapshot of the complete lipidome of egg yolks. Multivariate approaches like Principal Components Analysis (PCA) and Partial Least-Squares Discriminant Analysis (PLS-DA) were applied to screen and discover potential markers related to different types of eggs [10]. In another study, HILIC-TOF/MS enabled the analysis of phospholipids (PLs) in egg yolks from seven egg species (hen, pigeon, quail, duck, ostrich, emu, and goose) [11]. The relative quantities of several lipid classes were distinct among egg yolk types, and the study offered additional information about the nutritional value of eggs.

Lipidomic analysis has also been applied to estimate the impact of animal feeding methods on meat and egg quality [12]. Feed intervention can greatly alter the lipid content of eggs and meat, thus improving the nutritional value and health benefits of the products. Previous studies have reported differences in the lipidomic profile of eggs after using a modified feed in poultry diets with seal blubber oil [13] and different omega-3 polyunsaturated fatty acids [14]. More specifically, feeding different levels of seal blubber oil (SBO) for 5 weeks led to an important increase of PE, PC, and PI molecular species, but not Sph, nor LPC [13]. The increase of SBO in the diet, however, had less of an impact on PI. Furthermore, different n-3 PUFA-lipid profiles between regular eggs and eggs supplemented with n-3 PUFAs were found [14]. While synthesized or preformed docosahexaenoic acid (DHA) predominately existed in the glycerophospholipid form, dietary ALA was primarily deposited in the TG portion. Similarly, a HILIC-MS/MS lipidomics approach was applied, and significant variations in PL and TG profiles of eggs from hens fed with various diets (vegetable vs. animal) and raising environments (free range vs. indoor) were observed [15].

In the present study, an untargeted approach was applied to study the lipid profile of egg yolks from hens supplemented with xylanase; to the authors’ knowledge, the current study is the first to report differences in the lipidomic profile of egg yolks from hens with diets supplemented with xylanase. Different sample preparation methods and solvent mixtures were tested to optimise the extraction of total lipids, and a simple and cost-effective procedure was achieved compared to the existing methods in the literature. Multivariate statistical analysis of the data obtained in positive and negative ionisation modes provided signals for hundreds of lipids and highlighted differences in several egg yolk lipid species-classes. This work provides new data on egg lipidome, and in particular detailed lipid information on the lipid composition of egg yolk.

## 2. Materials and Methods

### 2.1. Chemicals and Reagents

LC-MS grade methanol (MeOH), dichloromethane (DCM), chloroform (CHCl_3_), methyl tert-butyl ether (MTBE), and ethanol ethyl acetate (AcOEt) used as extraction solvents were purchased from CHEM-LAB NV (Zedelgem, Belgium). Isopropanol (IPA) and acetonitrile (ACN) of LC-MS grade used as the mobile phase were also purchased from CHEM-LAB NV (Zedelgem, Belgium). LC-MS additives ammonium formate and formic acid (≥99%) were obtained from PANREAC QUIMICA SLU (Barcelona, Spain). A Milli-Q water purification system offered ultrapure water, 18.2 MΩ.cm (Millipore, Bedford, MA, USA).

### 2.2. Experimental Design and Housing of Animals

A total of 128 Isa Brown laying hens were divided into four treatments (A, B, C, D) and were housed in pairs in the experimental cages. Treatment A corresponded to the control group of hens following a normal diet. Treatments B, C, and D involved the addition of 10, 15, and 30 g/t of xylanase in the hens’ diets, respectively. Each treatment was replicated in 16 cage replicates. Each cage had dimensions of 41 × 41 cm, ensuring more available space per hen (840.5 cm^2^) compared to the directive from the EU (a minimum 750 cm^2^ of cage area per hen) [16]. The experimental layout and the level of enzyme supplementation in each treatment group are presented in Table S1.

The xylanase used is Xygest^TM^ HT (Kemi Industries Inc, Des Moines, IA, USA), a Thermopolyspora flexuosa-produced monocomponent xylanase that is expressed in Pichia pastoris and has intrinsic thermostability. Xygest HT has a minimal xylanase activity of 3,000,000 U/g on a corn starch-based carrier. Initially, the activity of xylanase in feed extract was estimated using an assay kit “Xylazyme AX” tablet test, as described by [17]. Table S1 summarizes the dosages and enzyme activity employed in the current experiment. The dietary ingredients and nutrients of the diets are shown in Table 1.

Feed samples were analysed for the NSP content and rest fiber fractions at the ILVO institute in Belgium (sample code = 2020/1576/99557| analysis date = 13 July 2020). Since NSP is composed of cell membranes and pectines, we determine NDF, a factor for cell membrane fractions, and we subtract NDF from NSP to obtain pectines. The fraction of the cell membranes was then split into hemicellulose, cellulose, and lignin by ADF and ADL determination. The difference of NDF—ADF is a measure for hemicellulose and the difference of ADF—ADL is a measure for cellulose. Table 1 summarises the results of the analysis.

### 2.3. Ethical Considerations

The study was approved by the research ethics committee of Aristotle University of Thessaloniki, Greece (approval number 71071/2020). The experimental conditions and procedures were in accordance with the requirements defined by Good Farming Practice Guidelines (Directive 2010/63/EC; Commission recommendation 2007/526/EC).

### 2.4. Sample Preparation

A thorough assessment was performed to study the effects of pre-treatment (lyophilisation), extraction solvent mixtures, and sample amounts as described in Section 3.2. Briefly, the egg white was manually removed from the fresh egg and the yolk was homogenized using an eggbeater. Afterwards, 50 mg of yolk were weighed in 1.5 mL Eppendorf tubes. For the lyophilized egg yolk samples, 25 mg of the sample were used, as the water content was calculated at 50% of the egg yolk.

During the lyophilisation procedure, fresh egg yolk samples were pre-frozen at −80 °C for 6 h and then lyophilised by a vacuum freeze-dryer. Drying temperature and the vacuum were set at −50 °C and 45 Torr, respectively, during the freeze-drying cycle. The duration of the freeze-drying process was 16 h. Finally, the lyophilised egg yolk powder was collected and stored at −20 °C.

The extraction of lipid content was performed by mixing 50 mg of dried sample with 1000 μL of MTBE: MeOH (5:1, *v*/*v*) and 1 min vortex. Centrifugation was performed for 30 min at 6700× *g* and supernatants (200 µL) were evaporated to dryness under the vacuum at room temperature using a speed vac concentrator (SpeedVac, Eppendorf Austria GmbH, Wien, Austria). Finally, reconstitution was performed by the addition of 400 μL water/acetonitrile/isopropanol (H_2_O/ACN/IPA) (1:1:3, *v*/*v*) followed by vortex to ensure thorough lipid dissolution.

### 2.5. Lipid Profiling Analysis (UHPC-TOF/MS)

For chromatographic analysis, a UHPLC Elute system was used and lipids were separated in a Waters ACQUITY UPLC CSH C18 column (2.1 mm × 100 mm × 1.7 μm, Waters Corp, USA). The mobile phase consisted of ACN:H_2_O (60:40, *v*/*v*) with 10 mM ammonium formate and 0.1% formic acid, and ACN:IPA (10:90, *v*/*v*) with 10 mM ammonium formate and 0.1% formic acid. The effective separation of lipids was achieved with the following gradient program at a flow rate of 0.4 mL/min: 60–57% A (0.0–2.0 min), 57–50% A (2.0–2.1 min), 50–46% A (2.1–12.0 min), 46–30% A (12.0–12.1 min), 30–1% A (12.1–18 min), 1–60% A (18.0–18.1 min), and 60% A (18.1–20.0 min). Column and autosampler temperatures were set at 55 and 8 °C, respectively. Injection volumes of 2 and 6 μL were used for positive and negative ionisation modes, respectively.

Mass spectrometer analysis was performed on the trapped ion-mobility time-of-flight (timsTOF) mass spectrometry system (Bruker, Bremen, DE) in positive and negative ionisation modes. Tuning parameters, for both positive and negative ESI, included capillary at ± 4.2 kV, dry gas 10 L/min, dry temperature at 200 °C, and nebulizer gas 2 Bar. For optimum ion transfer, Funnel RF 1 and 2 were set to 250 Vpp, Multipole RF at 200 Vpp, deflection delta at 80 V, transfer time at 54 µs, and pre-pulse storage 5 µs. Collision energy and RF were set to 10 eV and 1100.0 Vpp, respectively. A data-dependent acquisition mode was also applied within the MS method with an acquisition frequency of 6 (min) and 10 (max) Hz. Fragmentation was performed with a collision energy of 30 eV for all precursor ions. During the first segment of the analysis, 10 mM sodium formate was infused with MS with a flow rate 60,000 µL/h, and clusters were further used for internal calibration.

Before each analysis, blank samples (H_2_O: ACN: IPA 1:1:3 *v*/*v*) were injected into the system to assess system suitability. Quality control samples (QCs) were prepared by mixing equal volumes of each final reconstituted sample. QCs were analysed at the beginning and after every 5 real samples in order to equilibrate the system and assess its stability during analysis [12]. In addition, diluted QCs at different ratios (1:2, 1:4, 1:6, 1:8) in H_2_O: ACN: IPA (1:1:3, *v*/*v*/*v*)) were injected at the end of the analytical run to evaluate the dilution integrity of the detected features.

### 2.6. Data Processing and Chemometrics

Data were collected both as profile and centroid spectra. Initially, raw data recalibration was performed manually using Data Analysis Software (Bruker) and clusters of sodium formate. MSConvert (ProteoWizard 3.0.11567) online available free software was used for the conversion of the recalibrated centroid data to mzML that were further treated with XCMS (3.2.0) under R programming language. The extracted features were filtered and only those presented in 80% of QC samples and with CV <25% in QC samples were used for statistical analysis. Multivariate statistical analysis was performed by the SIMCA P software (version 13.0.2). Statistically significant features were assessed using a list with VIP (Variable Importance for the Projection), pcor, and *p*-Value from S-plots. For the significance of OPLS-DA models, statistical parameters such as R2X, R2Y (the fraction of the sum of squares for the selected component), Q2Y (the fraction of the total variation of Y), and the CV-ANOVA *p*-Value were examined. Significance of biomarkers was assessed by univariate statistical analysis using two-tailed students’ t-test and a threshold of *p* < 0.05 using Microsoft Excel. Finally, the log-ratio of lipids values between cases and controls (log2FC) was calculated in order to measure the changes due to different diets, and a threshold of log2FC > 0.5 was applied to the results.

Results from HRMS analysis were used for the annotation of statistically significant features. Using Reifycs Abf Converter (ver. 4.0.0), the raw LC-MS/MS data files were initially converted to .abf files. MS-DIAL (ver. 4.48) was used to process the data for peak selection, deconvolution of high energy spectra, feature alignment, and matching spectra from imported spectrum libraries (i.e., metabolite identification). Additionally, the accurate m/z values were matched to lipids using online available MS databases (Metlin, HMDB, and Lipidmaps). Then, fragmentation patterns from DDA experiments were assessed depending on the ability to obtain adequate signal, and spectra were compared to online available databases and the literature. Retention time and isotopic patterns were also considered before final structural elucidation. Level 2 identification was achieved for most of the statistically significant features, according to the identification level system of metabolites from untargeted analysis.

## 3. Results

### 3.1. UHPLC-TOF/MS Lipidomic Analysis

During the present study, we applied an advanced analytical platform to map the lipid profile of egg yolks after supplementation with xylanase. The ability of UPLC and the MS system to achieve increased peak capacity and scanning frequency allowed for the detection and identification of hundreds of lipids from various species in egg yolk samples. The CSH column allowed the elution and separation of lipid species with a broad range of polarities within 16 min in positive and negative ionisation modes, as illustrated in Figure S1. More specifically, polar lipids, such as LPC and LPE, eluted before 2.5 min, while neutral lipids, such as TG, eluted between 12.5 and 16 min.

### 3.2. Sample Preparation Optimisation

Lipid extraction is an important step in lipidomic analysis, and attention is required to avoid impurities from the matrix. To improve extraction efficiency, specific procedures must be adopted based on the sample matrix and target lipids. Liquid-liquid extraction (LLE) and solid phase extraction (SPE) are the methods of choice for the extraction of lipids from food and biological samples. LLE is the most frequently used approach in lipidomics due to its simplicity and high efficiency. The methods of Folch et al. and Bligh and Dyer [18,19] are often used for this purpose. However, a significant disadvantage of these extraction technologies when extracting lipids from the lower chloroform-rich layer is the increased risk of pollution from the upper layer and proteins at the interface between the two phases. To overcome this issue, Matyash et al. [20] introduced methyl tert-butyl ether (MTBE) as a lower-density organic solvent for lipid extraction to make the organic phase, and as a result, organic phase collection is easily achieved [21]. In this study, LLE was studied using the organic solvent mixtures DCM:MeOH (2:1, *v*/*v*), MTBE:MeOH (5:1, *v*/*v*), MTBE:AcOEt:MeOH (2:2:1, *v*/*v*/*v*), and CHCl_3_:MeOH (2:1, *v*/*v*), which have been used in literature [11,18,20,21] for the lipid extraction of egg yolk samples. In these mixtures, two phases (aqueous or aqueous-methanolic) are formed, and polar components of the egg yolk sample pass in the aqueous phase while lipids are transferred to the organic phase (ether or dichloromethane).

The results on the number and intensities of identified lipids were utilised to choose the optimal extraction solvent. In positive ionisation mode, nine lipid families were identified, including phosphatidylcholines (PC and PC O), sphingomyelins (SM), triacylglycerols (TG), diacylglycerols (DG), sterol lipids (ST), phosphatidylethanolamines (PE), ceramides (Cer), and lysophosphatidylethanolamines (LPE). In negative ionisation mode, 13 lipid families were identified, including phosphatidylcholines (PC and PC O), sphingomyelins (SM), phosphatidylethanolamines (PE), ceramides (Cer), lysophosphatidylethanolamine (LPE), phosphatidylglycerols (PG), phosphatidylinositols (PI), gangliosides (GM), N-acyl lysophosphatidylethanolamines (LNAPE), lysophosphatidylcholines (LPC), sterol lipids (ST), and phosphatidylethanolamines (PE-O).

**Figure 1:**
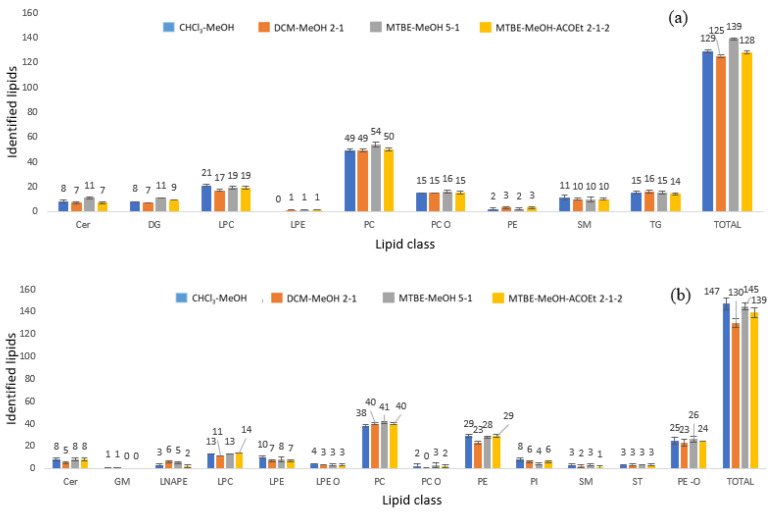
Number of lipids identified for each lipid family under different extraction solvents in (**a**) positive and (**b**) negative ionisation modes by triplicate analysis.

The effect of the lyophilisation process on the egg lipid content was also studied. The number of detected features and identified lipids was slightly higher in the fresh egg yolk samples for all the extraction solvent systems tested compared to those numbers found in the lyophilised egg yolk samples in both positive and negative ionisation modes, as shown in Figures S2 and S3. A total amount of 261 lipids were detected in the MTBE: MeOH (5:1, *v*/*v*) extraction method for the lyophilised egg yolk and 284 lipids were found in fresh egg yolk samples in positive and negative ionisation mode (aggregate of the two modes). With CHCl_3_: MeOH (2:1, *v*/*v*) extraction, 257 lipids were identified for the lyophilised egg yolk, and 276 lipids were found in fresh egg yolk samples. A total number of 257 and 255 lipids were identified in lyophilized and fresh egg yolk, respectively, whereas in MTBE: AcOEt: MeOH (2:2:1, *v*/*v*/*v*), 249 lipid compounds were identified for the lyophilised egg yolk and 267 lipids for the fresh egg yolk in negative and positive ionisation modes. Since lyophilisation did not lead to more efficient lipid extraction, which is in accordance with the results from our previous study [22], fresh egg yolks were further used for lipid profiling assessment. Comparing the different extraction protocols on fresh egg yolks, we observed that the number of identified lipids is moderately dependent on the solvent system used, although all lipid classes were observed by applying the different extraction conditions. With MTBE:MeOH (5:1, *v*/*v*) we were able to detect a slightly higher number of lipids compared to the other extraction solvents; thus, it was selected for the present study. It is important to mention that DCM:MeOH (2:1, *v*/*v*) did not favour the extraction of diacylglycerols and ceramides compared to the other organic mixtures, although the mixture assisted with the extraction of phosphoinositol and N-acylphosphatidylethanolamines. With regards to lipid composition, the distribution of the major lipid groups detected is shown in Figure 1. Glycerophospholipids were found to be the predominant lipid group for all the extraction solvent systems tested, especially phosphatidylcholines (PC) and phosphatidylethanolamines (PE). This observation is in alignment with previous reports [23,24,25].

### 3.3. Lipidomic Profiling

For investigations based on lipidomics, instrument stability is important for effective and precise retention time alignment and peak annotation. As a result, the recognized and established QC criteria indicated above were used to filter the identified features. A total of 6806 and 2209 ions in positive and negative mode met the quality control criteria and were selected for further data analysis from the derived lipid profiles. All samples, including QCs, were subjected to unsupervised PCA, and as shown in Figures S4 and S5, the QC samples are closely grouped to the centre of the plot, confirming the analytical system’s suitability and reproducibility in both ionisation modes.

**Figure 2:**
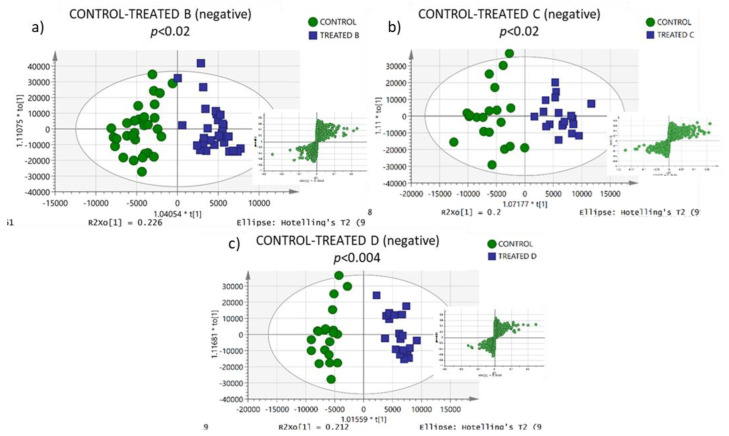
OPLS-DA models of (**a**) control and treated-b, (**b**) control and treated-c, and (**c**) control and treated-d group samples in negative ionisation model. Ions contributing to the differentiation of the groups are located at the edges of the S-plot.

**Figure 3:**
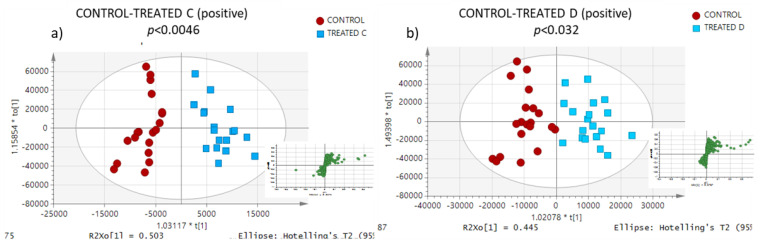
OPLS-DA models of (**a**) control and treated-c, and (**b**) control and treated-d group samples in positive ionisation mode. Ions contributing to the differentiation of the groups are located at the edges of the S-plot.

Additionally, clear discrimination can be observed in OPLS-DA models (Figure 2 and Figure 3) between egg yolks from hens after feed intervention with xylanase (control vs. treated groups B, C, and D) in both ionisation modes. Results from permutation analysis showed that the OPLS-DA models were robust with high predictability. An essential evaluation of the OPLS models was conducted using cross-validation ANOVA testing (CV-ANOVA) (*p* < 0.05 for both positive and negative ionisation modes). The significance of the multivariate models resulted in the discovery of highly significant features.

**Figure 4:**
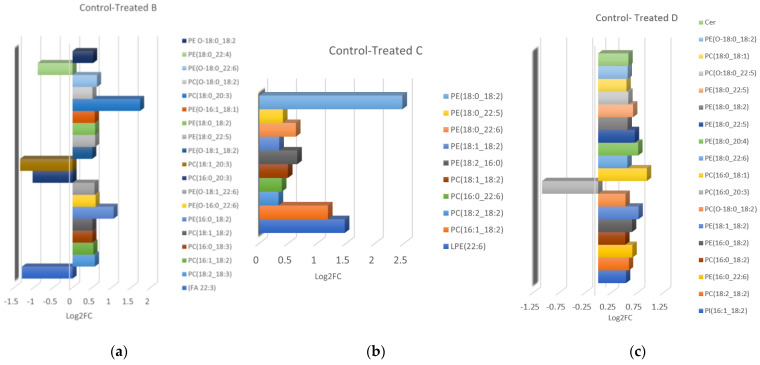
Increased (positive values) or decreased (negative values) annotated lipids found in (**a**) treated-b, (**b**) treated-c, and (**c**) treated-d group samples in negative ionisation mode.

**Figure 5:**
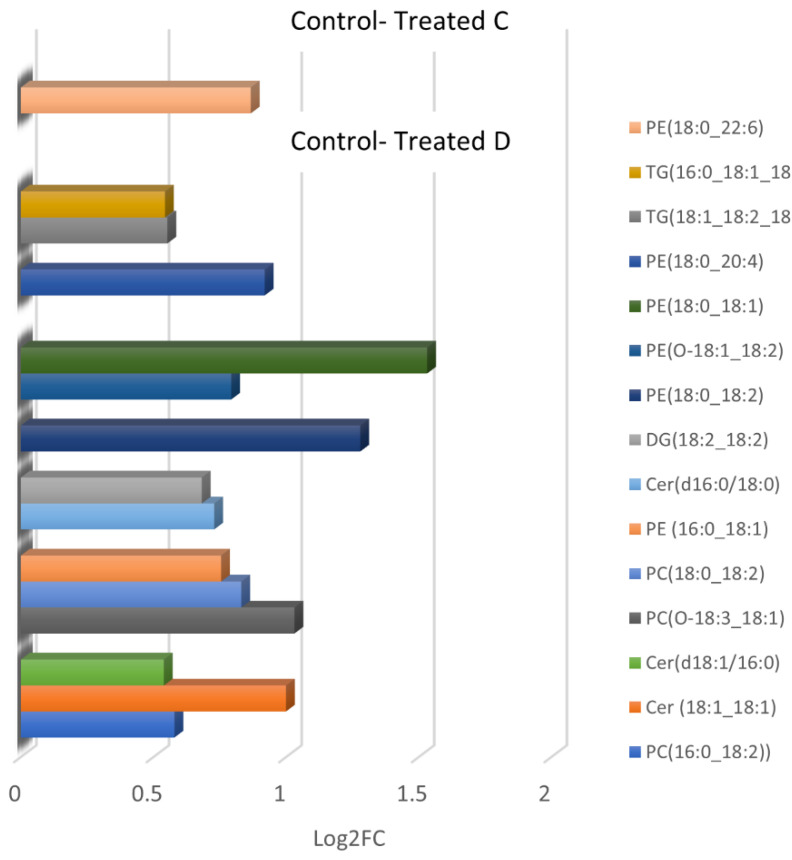
Identified lipids that were found to be increased in the egg yolks of treated c and treated d groups in positive ionisation mode.

Lipid identification was based on isotopic pattern and spectral matching (>80%) using MS-DIAL with a prerequisite of mass accuracy being below 5 ppm. Lipid identification was performed depending on head groups and lipid species. Most phospholipids were identified as distinct adducts: [M + H]^+^ or [M + NH_4_]^+^ in positive mode and [M–H]^-^ or [M + HCOO]^-^ in negative mode. In positive mode, neutral lipids such as TGs and DGs were identified as NH_4_ adducts. However, the fatty acid contents of phospholipids (PLs) are only determined in negative ion mode, whereas head group and/or neutral loss are determined in positive ion mode [26]. In total, 23 (control vs. treated group b), 13 (control vs. treated group c), and 20 features (control vs. treated group d) were statistically significant (*p*-Value < 0.05) in negative ionisation mode after applying a series of evaluation criteria S-plots with *p* ≥ |0.05|, *p*(corr) ≥ |0.5|, and VIP > 1. Table 1 shows the significant features and annotated lipids (MS Level 2) found, as described in Section 2.6. Phosphatidylcholines (PC and PC O), phosphatidylethanolamines (PE and PE O), phosphatidylinositols (PI), and a fatty acid (FA) exhibited important differences between the studied groups (control vs. treated). The identified lipids found upregulated or downregulated in the different groups are shown in Figure 4. In positive ionisation mode, lipids belonging to the groups PC and PC O, PE and PE O, TG, DG, and Cer were discovered to be discriminated (Table 2). All significant annotated lipids were found to increase in the treated groups (Figure 5).

Overall, the CSH column provided chromatographic selectivity and separation power favoring lipidomics analysis. Combination with high-resolution MS and MS/MS detection enabled the annotation of numerous lipids covering different classes and polarities by a combination of either automatic and/or manual data manipulation and library searching. In Figure S6, a manually annotated phosphatidylethanolamine is depicted.

## 4. Discussion

A wide variety of necessary lipids found in egg yolks play structural and functional roles in membranes during cell signaling [27]. Egg yolk is the main source of phospholipids in nature, and it is estimated that it consists of 33% PLs, 62% TGs, and 5% cholesterol [23]. PLs are a unique class of chemical molecules found in all living creatures as key components of cellular membranes (lipid bilayers) and as a source of condensed energy [28]. Phospholipids also lower triglyceride and cholesterol levels in the blood, as well as prevent cholesterol from accumulating in the artery wall. Ultimately, PLs play an important role in cell proliferation and cell death signaling. Furthermore, phospholipid metabolism is involved in several human disorders, including cardiovascular and cancer diseases, Alzheimer’s and Parkinson’s diseases, and diabetes [28]. Phosphatidylcholine (PC), phosphatidylethanolamine (PE), sphingomyelin (SM), lysophosphatidylcholine (LPC), lysophosphatidylethanolamine (LPE), and phosphatidylglycerol (PG) are all found in egg yolk [29], and PC (73%) and PE (15.5%) constitute the most prominent phospholipid fractions.

The present study is the first to report differences in the lipidomic profile of egg yolks from hens’ diets enriched with various amounts of xylanase. UHPLC-TOF/MS untargeted lipidomic analysis revealed a significantly altered PL profile in egg yolks from hens after dietary supplementation of xylanase. PC, a subclass of phospholipids, is commonly found in living organisms and plays a vital role in delivering sustenance and protection to the body [30]. Among the statistically significant features in negative ESI, 8, 4, and 7 molecular species of PC were identified after the addition of 10, 15, and 30 g/t of xylanase in hens’ diets, respectively. Egg yolk PE were found to show high antioxidant activity and may have positive effects on human health [31]. Here, 9, 6, and 9 PE species were found to increase in egg yolk samples after 10, 15, and 30 g/t (groups B, C, and D) of xylanase supplementation, indicating the potential added value in samples after treatment. It is notable that two PE species, PE(18:0_22:5) and PE(O-18:0_18:2), were identified as significant lipids between all treated groups versus the control, while PE(18:0_18:2) was found to differ significantly between the control and the treated groups B and C. The results in positive ESI indicated that feed intervention with 10 g/t of xylanase did not show any statistically significant changes in the lipid profile of egg yolks. However, feeding higher levels of xylanase (C, D groups) resulted in a significant increase of PE, PE O, PC, PC O, Cer, DG, and TG molecular species, with PE and PC being again the most abundant lipid families. All significant lipids were found increased in treated egg yolk samples. The above observations are consistent with previous reports investigating xylanase supplementation on performance, egg quality, and nutrient digestibility in laying hens that are fed wheat-based diets [32,33,34]. Additionally, studies investigating changes of yolk lipid composition during storage have suggested PLs, TG, and DG as markers of quality [35,36,37]. More specifically, TGs, DGs, and most PLs were found to increase during storage, and to indicate decreased quality.

To our knowledge, there is not much evidence on the potential effects of dietary xylanase supplementation on quality characteristics of egg yolks in laying hens. Papadopoulos et al. [17] showed that xylanase supplementation in wheat-based diets in laying hens could improve egg yolk color at all supplemented levels compared to the control diet. Meanwhile, xylanase supplemented at the higher level (90,000 U/g) improved polyunsaturated and reduced monounsaturated egg yolk fatty acid content. Since eggs are of the most popular and affordable functional foods, these findings are promising, since one of the major problems facing the food industry is consumer health. Our results indicate that xylanase supplementation in hens’ diets led to a significant increase of lipids with high antioxidant value playing important roles in body protection. More research should be done to examine the effectiveness of xylanase in altering yolk lipid content in various feed ingredients. Producing eggs with improved nutritional characteristics at a reduced price is likely to appeal to consumers.

Overall, utilisation of high-resolution mass spectrometry can lead to potential candidate elemental composition of molecular and product ions, thus promoting lipid identification; in addition, chromatographic separation results in decreased ion suppression for mass spectrometric detection, adding an orthogonal (to MS) separation mechanism [38]. In the current study, a significant number of lipid species were identified in control and treated egg yolk samples. The proposed lipidomics approach is rapid, robust, and effective for discriminating egg yolks from hens fed with modified diets. The current lipidomics protocol provides a wealth of new information on egg lipid content. Further research is needed to better understand the relationships between egg yolk lipid profiles and hens’ supplied diets and underlying mechanisms.

## 5. Conclusions

Untargeted lipidomic profiling by UHPLC-TOF/MS was applied to evaluate a comprehensive landscape of lipid species in egg yolk samples. A total of 6806 and 2209 ions, in negative and positive mode, respectively, were chosen for additional data analysis because they satisfied the quality control (QC) requirements (RSD 30%). Multivariate and univariate statistical analysis resulted in the discovery and identification of highly significant features/lipids. Tight clustering of QC samples in PCA models confirmed the stability, suitability, and repeatability of the analytical system. The constructed OPLS-DA models revealed classification between egg yolks from hens’ fed diets supplied with xylanase (treated groups B, C, and D) and the control ones (group A). This study provides a thorough elucidation of the lipidomic changes related to various stages of xylanase supplementation in laying hen diets and potential contributions to egg quality.

## Figures and Tables

**Table 1:** Main ingredients and nutrient analysis of the diets.

Ingredient (Labeling Information)	kg	%
Wheat soft	55.03	55.03
Soybean meal (47%)	15.40	15.40
Sunflo.Meal34	10.00	10.00
Limestone	9.08	9.08
Corn, Yellow Lys	5.00	5.00
Soybean oil	3.73	3.73
Mcp	0.55	0.55
Mineral and vitamin premix a	0.40	0.40
Sodium chloride	0.29	0.29
D,L-Methionine	0.16	0.16
L-Lysine Hcl	0.15	0.15
Phytase 500	0.06	0.06
Sodium bicarbonate	0.05	0.05
Pigment red	0.04	0.04
L.Threonine	0.04	0.04
L-Valine	0.02	0.02
Total	100.00	100.00
Provision-Nutrient Analysis	UM	%AF
Dry matter	%	89.00
Crude Protein	%	17.50
Crude Fat	%	4.99
Crude Fiber	%	3.99
NSP	%	14.88
Starch	%	35.83
Ca	%	3.90
Phosphorus	%	0.53
Digestible Phosphorus	%	0.40
Na	%	0.17
Cl	%	0.25
K	%	0.73
Lysine dig	%	0.76
Methionine dig	%	0.41
Cystine dig	%	0.26
Methionine + Cystine dig	%	0.67
Tryptophan dig	%	0.18
Threonine dig	%	0.53
Ile dig	%	0.61
Arginine dig	%	1.00
Valine dig	%	0.70
Metabolizable energy (ABE poultry based on CVB)	Kcal/	2700.00

^a^ Provided per kg diet: Retinyl acetate: 4.2 mg; Cholecalciferol: 0.1 mg; α- tocopherol acetate: 31.25 mg; Menadione: 5.0 mg; Cyanocobalamin: 0.025 mg; folic acid: 1.0 mg; Choline chloride:: 450 mg; Pantothenic acid: 12.5 mg; Riboflavin, 6.25 mg; Nicotinic acid: 43.75 mg; Thiamin: 3.0 mg; D-biotin: 0.1 mg; Pyridoxine: 5.0 mg; Manganese: 125 mg; Zinc: 112 mg; Iron: 62 mg; Copper: 10 mg; Iodine: 1.0 mg; Selenium: 0.15 mg.

**Table 2:** Statistically significant features in egg yolks of groups B, C, and D in comparison with the control (group A) with annotation, *p*-Value, and log2fold change.

A/A	VIP	*m/z*	RT min	*p*-Value	Log2FC	Annotation	MS DIAL	Mass Error (ppm)
-ESI								
CONTROL-TREATED B								
1	3.20	305.2497	2.72	5.28 × 10^−3^	−1.30	FA 20:3	FA 20:3	3.60
2	1.14	824.5457	4.76	2.84 × 10^−2^	0.57	PC(18:2_18:3)	PC(18:2_18:3)	1.21
3	7.10	800.5449	5.39	4.52 × 10^−2^	0.53	PC(16:1_18:2)	PC(16:1_18:2)	0.25
4	3.15	801.5453	5.67	3.25 × 10^−2^	0.51	PC(16:0_18:3)	PC(16:0_18:3)	-
5	4.05	828.5751	7.03	4.07 × 10^−3^	0.51	PC(18:1_18:2)	PC(18:1_18:2)	−1.08
6	4.93	714.5084	7.42	4.61 × 10^−2^	1.05	PE(16:0_18:2)	PE(16:0_18:2)	0.69
7	1.40	776.5310	7.57	1.17 × 10^-^³	−0.92	Unknown	-	-
8	3.62	748.5285	7.79	1.49 × 10^−2^	0.59	PE(O-16:0_22:6)	PE(O-16:0_22:6)	0.26
9	1.67	774.5466	7.94	2.21 × 10^−2^	0.56	PE(O-18:1_22:6)	PE(O-18:1_22:6)	2.96
10	5.31	828.5767	8.04	6.89 × 10^−3^	−1.02	PC(16:0_20:3)	PC(16:0_20:3)	0.84
11	2.06	854.5923	8.26	3.18 × 10^−3^	−1.35	PC(18:1_20:3)	PC(18:1_20:3)	0.70
12	1.94	726.5449	9.03	2.14 × 10^−2^	0.50	PE(O-18:1_18:2)	PE(O-18:1_18:2)	−0.82
13	1.88	792.5543	9.55	6.71 × 10^−3^	0.58	PE(18:0_22:5)	PE(18:0_22:5)	−0.75
14	1.23	661.5068	9.63	3.06 × 10^−3^	0.99	Unknown	-	-
15	3.23	742.5390	9.83	4.09 × 10^−2^	0.58	PE(18:0_18:2)	PE(18:0_18:2)	−0.26
16	4.64	700.5279	10.53	2.83 × 10^−2^	0.57	PE(O-16:1_18:1)	PE(O-16:1_18:1)	−0.99
17	6.97	856.6116	10.65	5.28 × 10^−4^	1.73	PC(18:0_20:3)	PC(18:0_20:3)	5.01
18	1.77	816.6139	10.71	2.79 × 10^−2^	0.51	PC(O-18:0_18:2)	PC(O-18:0_18:2)	1.83
19	1.62	686.5746	10.73	7.90 × 10^−5^	1.41	Unknown	-	-
20	1.10	777.565	10.77	2.83 × 10^−3^	0.63	PE(O-22:6_18:0)	PE(O-18:1_22:5)	-
21	1.19	794.5709	11.46	1.64 × 10^−2^	−0.89	PE(18:0_22:4)	PE(18:0_22:4)	0.50
22	4.06	728.5594	11.69	5.88 × 10^−3^	0.53	PE(O-18:0_18:2)	PE(O-18:0_18:2)	−0.68
23	1.26	858.624	12.43	5.77 × 10^−3^	1.76	Unknown	-	-
CONTROL-TREATED C								
1	3.36	524.2792	1.07	1.20 × 10^−3^	1.47	LPE 22:6	LPE 22:6	1.71
2	6.19	800.5446	5.39	4.64 × 10^−3^	1.18	PC(16:1_18:2)	PC(16:1_18:2)	−0.12
3	6.47	826.5611	5.70	1.36 × 10^−3^	0.53	PC(18:2_18:2)	PC(18:2_18:2)	0.84
4	5.24	762.5086	6.59	9.49 × 10^−4^	0.65	PE(16:0_22:6)	PE(16:0_22:6)	0.91
5	5.05	828.5761	7.04	4.85 × 10^−4^	0.50	PC(18:1_18:2)	PC(18:1_18:2)	0.12
6	7.24	714.5083	7.42	6.27 × 10^−3^	0.65	PE(16:0_18:2)	PE(16:0_18:2)	0.55
7	1.31	776.5310	7.57	1.27 × 10^−4^	1.06	Unknown	-	-
8	1.87	740.5239	7.63.	3.18 × 10^−3^	0.54	PE(18:1_18:2)	PE(18:1_18:2)	0.40
9	4.40	790.5400	8.74	4.21 × 10^−3^	0.63	PE(18:0_22:6)	PE(18:0_22:6)	1.01
10	1.52	898.5817	9.09	5.91 × 10^−3^	−0.66	Unknown	-	
11	1.84	792.5543	9.55	5.45 × 10^−3^	0.51	PE(18:0_22:5)	PE(18:0_22:5)	−0.75
12	5.95	742.5391	9.84	1.76 × 10^−3^	2.47	PE(18:0_18:2)	PE(18:0_18:2)	−0.13
13	1.45	686.5746	10.73	4.79 × 10^−5^	0.85	Unknown	-	-
CONTROL-TREATED D								
1	12.13	540.3319	1.32	1.08 × 10^−2^	0.51	LPC(16:0)	-	2.22
2	2.79	833.5183	4.98	4.88 × 10^−3^	0.53	PI(16:0_18:2)	PI(16:0_18:2)	−0.35
3	5.51	826.5616	5.70	2.78 × 10^3^	0.59	PC(18:2_18:2)	PC(18:2_18:2)	1.45
4	5.29	762.5087	6.58	3.04 × 10^−4^	0.66	PE(16:0_22:6)	PE(16:0_22:6)	1.04
5	12.19	802.5607	6.84	1.49 × 10^−3^	0.52	PC(16:0_18:2)	PC(16:0_18:2)	0.37
6	7.58	714.5083	7.42	1.15 × 10^−3^	0.65	PE(16:0_18:2)	PE(16:0_18:2)	0.55
7	5.42	740.5239	7.62	2.63 × 10^−4^	0.77	PE(18:1_18:2)	PE(18:1_18:2)	0.40
8	1.68	816.5766	7.85	1.34 × 10^−3^	0.52	PC(O-18:0_18:2)	PC(17:0_18:2)	-
9	1.88	828.5771	8.04	8.51 × 10^−4^	−1.08	PC(16:0_20:3)	PC(16:0_20:3)	1.32
10	1.58	804.5770	8.48	2.19 × 10^−3^	0.93	PC(16:0_18:1)	PC(16:0_18:1)	1.24
11	3.79	790.5408	8.74	4.94 × 10^−3^	0.56	PE(18:0_22:6)	PE(18:0_22:6)	2.02
12	7.94	766.5388	9.43	9.60 × 10^−3^	0.77	PE(18:0_20:4)	PE(18:0_20:4)	−0.52
13	1.71	792.5555	9.54	4.47 × 10^−3^	0.70	PE(18:0_22:5)		0.75
14	1.37	661.5068	9.62	5.74 × 10^−5^	0.83	Unknown	-	-
15	8.21	742.5391	9.83	7.59 × 10^−4^	0.56	PE(18:0_18:2)	PE(18:0_18:2)	−0.13
16	4.32	793.5746	10.47	1.43 × 10^−3^	0.67	PE(18:0_22:5)	-	0.88
17	1.46	776.5607	10.77	3.12 × 10^−3^	0.58	PC(O:18:0_22:5)	PC(O:18:0_22:5)	-
18	16.08	832.6080	11.17	6.65 × 10^−3^	0.54	PC(18:0_18:1)	PC(18:0_18:1)	0.84
19	3.70	728.5599	11.68	2.28 × 10^−3^	0.57	PE(O-18:0_18:2)	PE(O-18:0_18:2)	0.00
20	1.39	722.6678	14.85	4.22 × 10^−4^	0.58	Unknown Cer	Cer 29:0;2O/15:1	-
CONTROL-TREATED C								
1	2.11	792.5495	8.80	1.40 × 10^−3^	0.86	PE(18:0_22:6)	PE(18:0_22:6)	−5.29
2	1.73	600.4943	9.82	4.60 × 10^−3^	0.99	Unknown		-
CONTROL-TREATED D								
1	9.79	758.5695	6.92	2.03 × 10^−3^	0.58	PC(16:0_18:2)		0.13
2	1.99	564.5344	7.47	1.31 × 10^−4^	1.00	Cer(d18:1_18:1)		−1.06
3	0.66	738.4893	7.48	5.63 × 10^−4^	0.83	Unknown		-
4	0.87	520.5087	8.78	6.12 × 10^−3^	0.54	Cer(d18:1_16:0)		−1.34
5	1.70	793.5402	8.80	7.09 × 10^−5^	1.03	PC(O-18:3_18:1)		-
6	10.03	786.6009	9.15	1.19 × 10^−3^	0.83	PC(18:0_18:2)	PC(18:0_18:2)	0.25
7	1.11	718.5372	9.30	5.62 × 10^−3^	0.75	PE(16:0_18:1)	PE (18:1_16:0)	−1.25
8	0.89	540.5357	9.67	6.63 × 10^−3^	0.73	Cer(d18:0_16:0)	Cer(d18:0/16:0)	1.29
9	1.42	634.5319	9.81	1.01 × 10^−3^	0.68	DG(18:2_18:2)	-	2.20
10	0.80	599.4921	9.82	9.73 × 10^−4^	0.71	Unknown	-	-
11	0.90	617.5026	9.82	6.37 × 10^−4^	0.88	Unknown	-	-
12	2.49	744.5537	9.88	2.63 × 10^−3^	1.28	PE(18:0_18:2)	PE(18:0_18:2)	−0.13
13	0.74	728.5544	11.30	2.55 × 10^−3^	0.79	PE(O-18:1_18:2)	PE(O-18:1_18:2)	−6.03
14	2.47	746.5712	12.24	3.50 × 10^−3^	1.53	PE(18:0_18:1)	-	2.41
15	1.04	768.5539	12.26	1.40 × 10^−3^	0.92	PE(18:0_20:4)	-	0.13
16	0.70	896.7705	15.74	9.32 × 10^−4^	0.55	TG(18:1_18:2_18:3)	-	0.33
17	2.92	874.7860	15.95	1.17 × 10^−3^	0.54	TG(16:0_18:1_18:2)	TG(16:0_18:1_18:2)	0.22

## Data Availability

Data is presented within the article.

## Supplementary Materials

The following supporting information can be downloaded at: https://www.mdpi.com/article/10.3390/metabo13050649/s1, Table S1. Level of supplementation and enzyme activities used in the present study.,., Figure S1: Overlaid extracted ion chromatograms of lipids present in a QC sample from different classes in (a) positive and (b) negative ESI., Figure S2: Number of identified lipids for lyophilised and non-lyophilised samples by triplicate analysis extracted with four different solvents in negative ionisation mode. Total numbers are given in the inset. Figure S3: Number of identified lipids for lyophilised and non-lyophilised samples by triplicate analysis extracted with four different solvents in positive ionisation mode. Total numbers are given in the inset., Figure S4: Principal component analysis (PCA) score plot of samples in negative ionisation mode showing the QC samples clustered in the centre of the plot., Figure S5: Principal component analysis (PCA) score plot of samples in positive ionisation mode showing the QC samples clustered in the centre of the plot., Figure S6: Chromatographic peak of phosphatidylethanolamine (PE O-18:0 18:2) together with MS and MS/MS spectrum. Precursor and MS fragments facilitated peak annotation.
