# Supplementary material for: Untargeted UHPLC-TOF/MS Lipidomic Analysis for the Investigation of Egg Yolks after Xylanase Supplementation of the Diet of Laying Hens
Source: Metabolites. 2023 May 10;13(5):649. doi: 10.3390/metabo13050649 (PMC10224551; doi:10.3390/metabo13050649)
Supplement: Supplementary file 1 [file metabolites-13-00649-s001.zip › metabolites-2309631-supplementary.pdf]

Untargeted UHPLC-TOF/MS lipidomic analysis for the investigation of egg yolks after xylanase supplementation of the diet of laying hens

Table S1. Level of supplementation and enzyme activities used in the present study.

Treatment	Level of supplementation	Intended Activity	Batch Corrected Activity	Recovered Activity in Feed
T1 control	0 g/t	0 U/kg	0 U/kg	0 U/kg
T2	10 g/t	30,000 U/kg	41,058 U/kg	35,861 U/kg
T3	15 g/t	45,000 U/kg	61,587 U/kg	57,451 U/kg
T4	30 g/t	90,000 U/kg	123,174 U/kg	111,936 U/kg

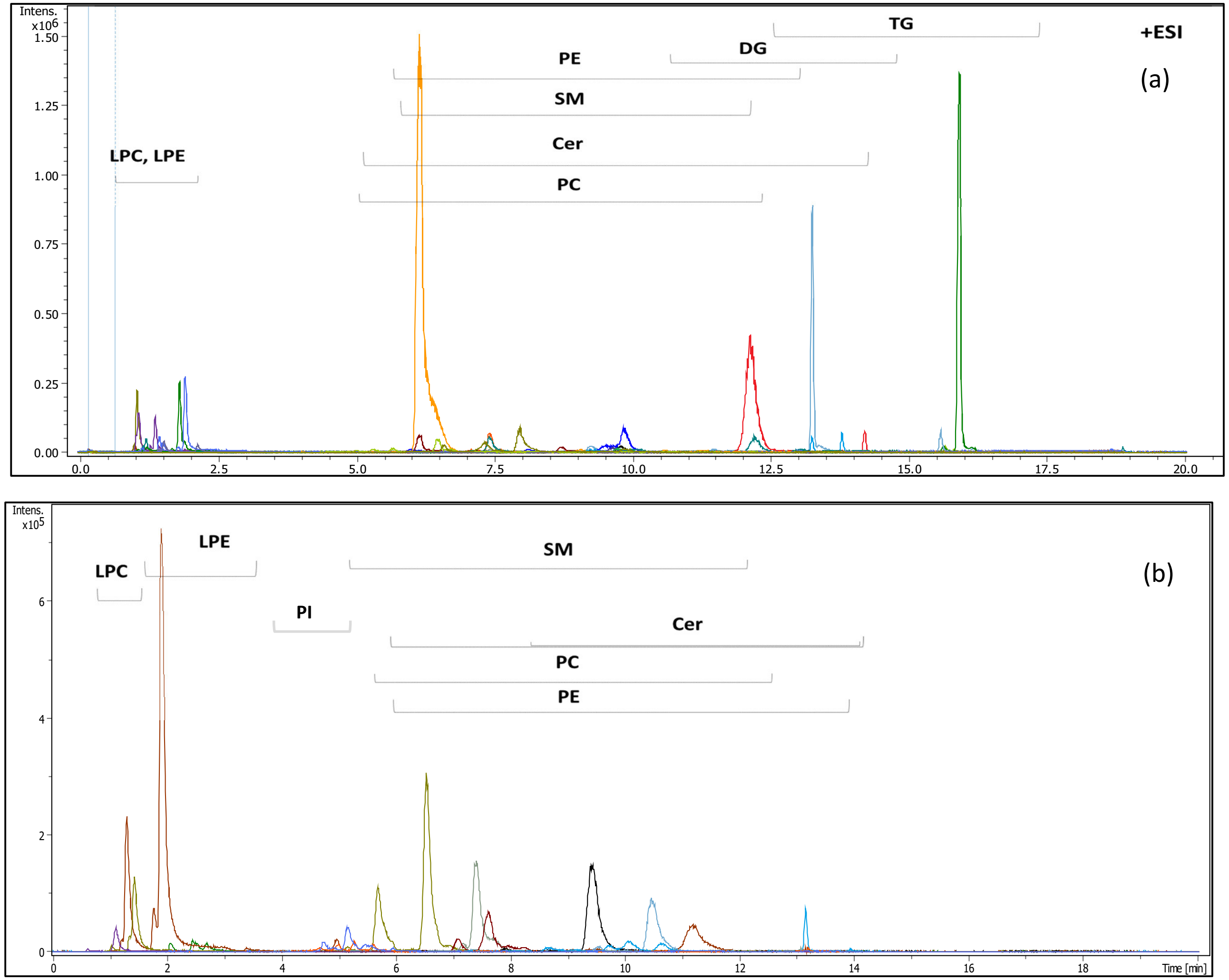

Figure S1: Overlaid extracted ion chromatograms of lipids present in a QC sample from different classes in (a) positive and (b) negative ESI.

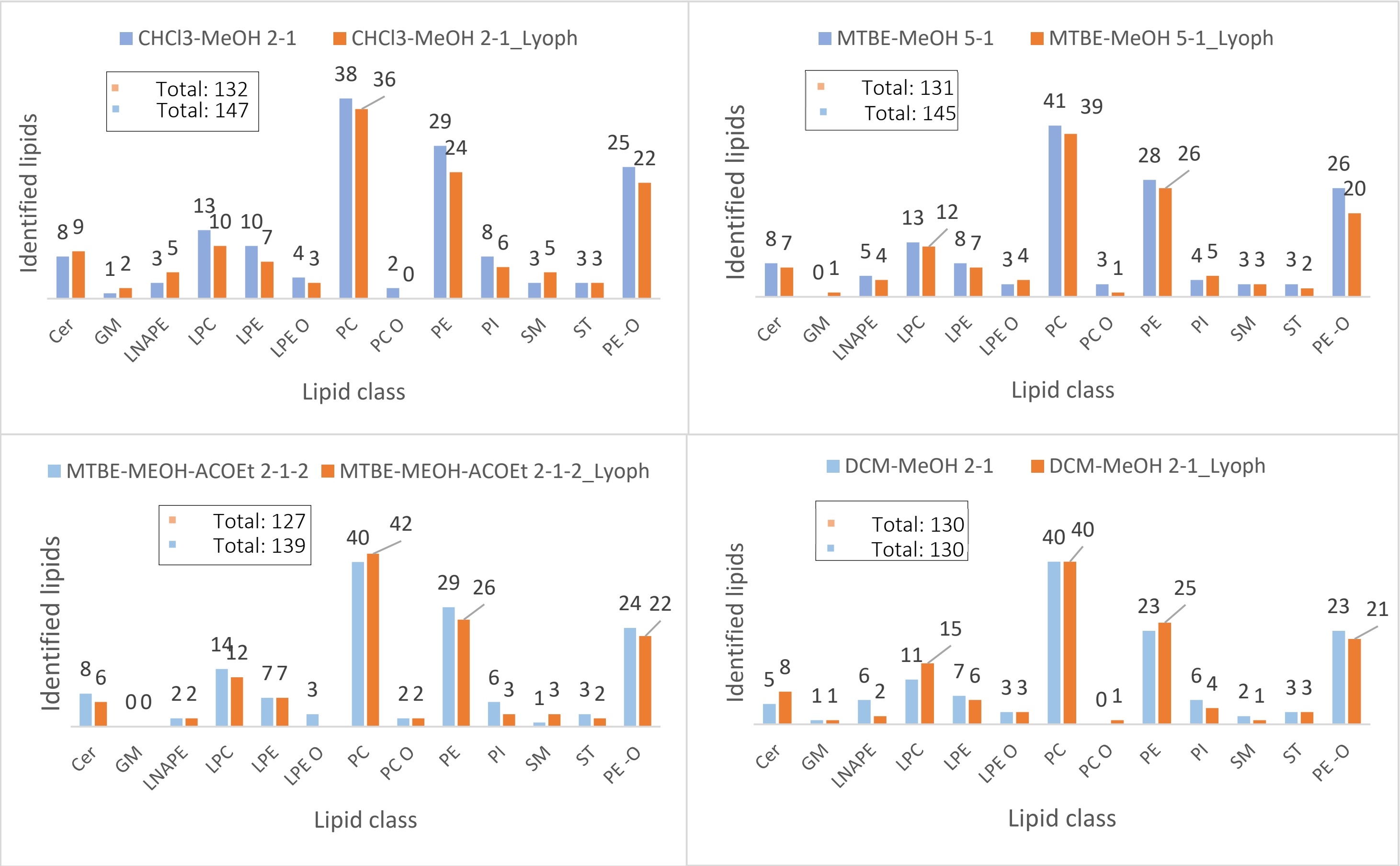

Figure S2: Number of identified lipids for lyophilised and non-lyophilised samples by triplicate analysis extracted with four different solvents in negative ionisation mode. Total numbers are given in the inset.

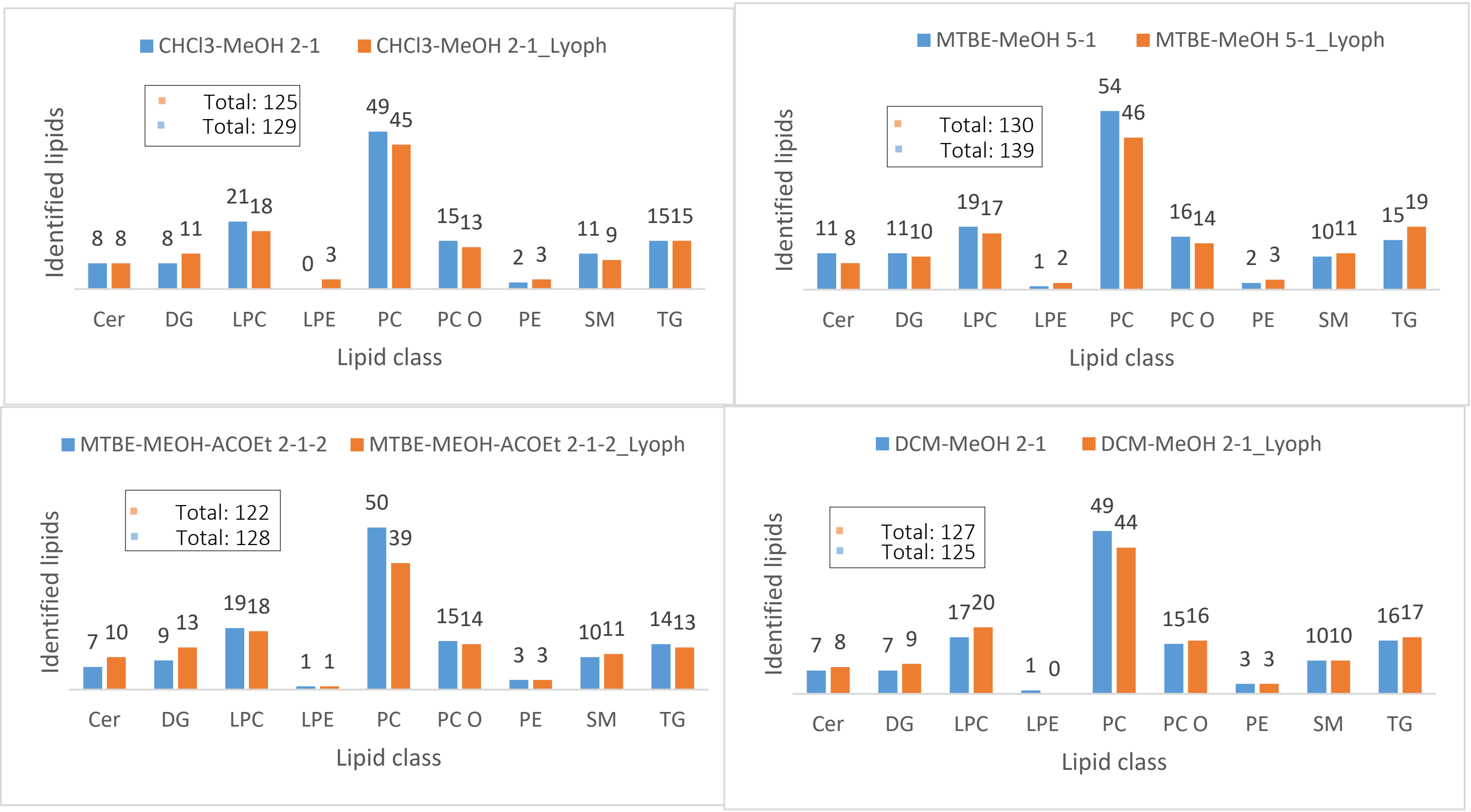

Figure S3: Number of identified lipids for lyophilised and non-lyophilised samples by triplicate analysis extracted with four different solvents in positive ionisation mode. Total numbers are given in the inset.

.

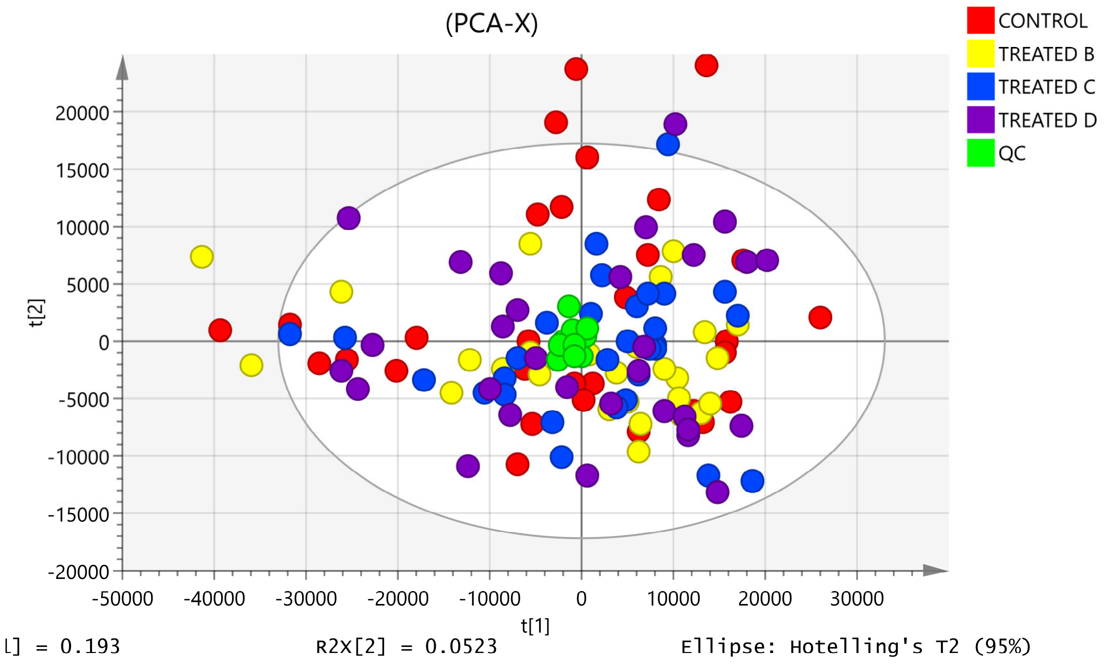

Figure S4: Principal component analysis (PCA) score plot of samples in negative ionisation mode showing the QC samples clustered in the centre of the plot.

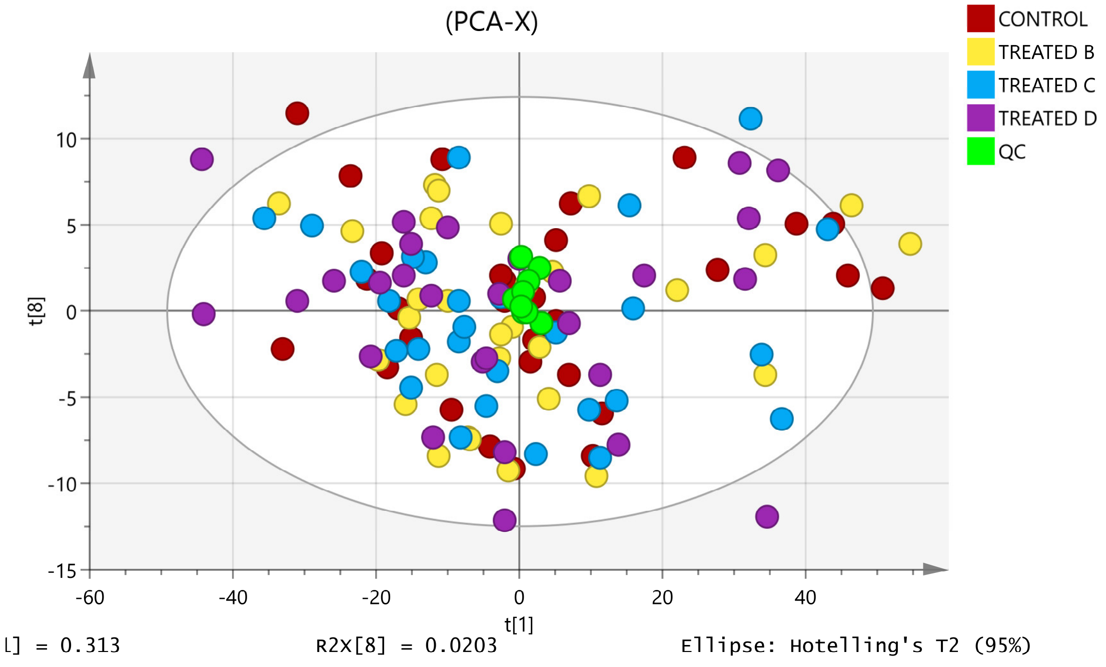

Figure S5: Principal component analysis (PCA) score plot of samples in positive ionisation mode showing the QC samples clustered in the centre of the plot.

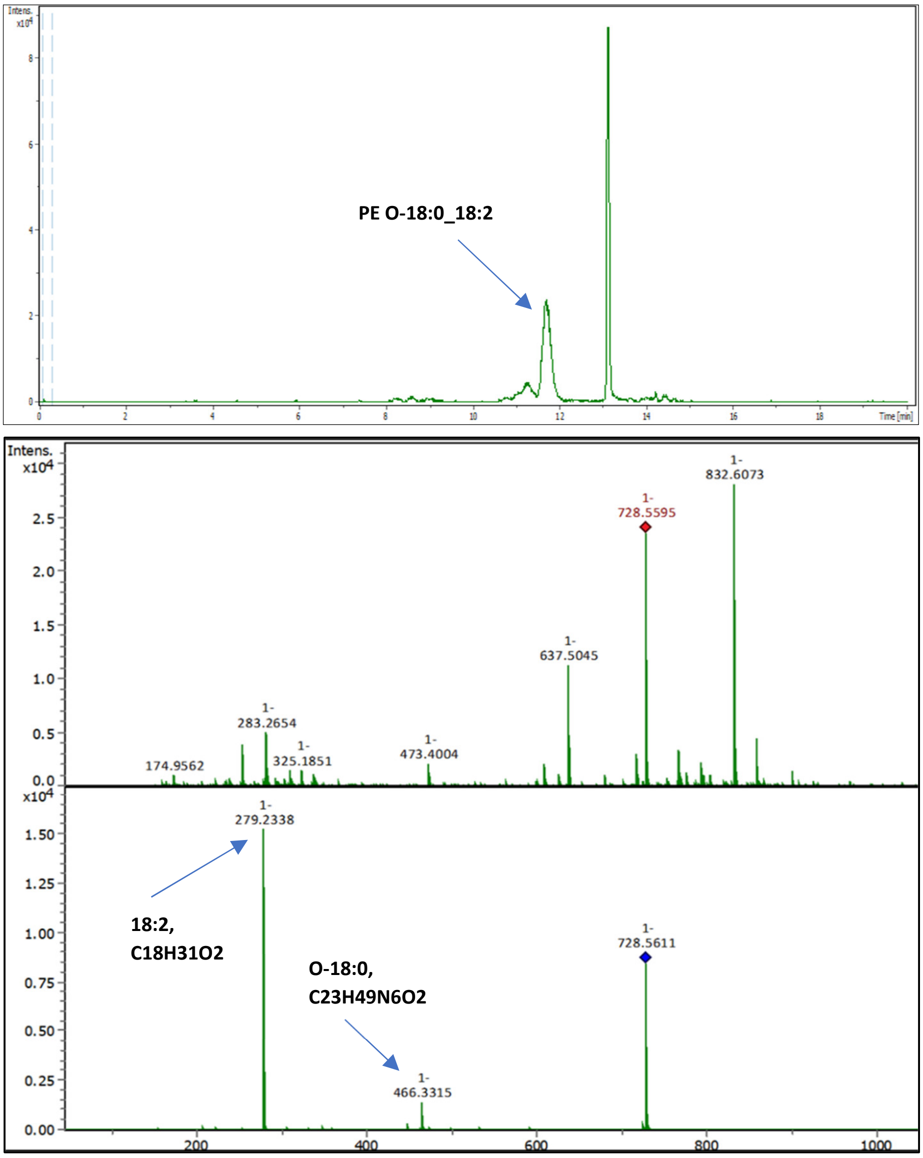

Figure S6: Chromatographic peak of phosphatidylethanolamine (PE O-18:0 18:2) together with MS and MS/MS spectrum. Precursor and MS fragments facilitated peak annotation**.**
